# Applications of large language models in psychiatry: a systematic review

**DOI:** 10.3389/fpsyt.2024.1422807

**Published:** 2024-06-24

**Authors:** Mahmud Omar, Shelly Soffer, Alexander W. Charney, Isotta Landi, Girish N. Nadkarni, Eyal Klang

**Affiliations:** ^1^ Faculty of Medicine, Tel-Aviv University, Tel-Aviv, Israel; ^2^ Internal Medicine B, Assuta Medical Center, Ashdod, Israel; ^3^ Ben-Gurion University of the Negev, Be’er Sheva, Israel; ^4^ Icahn School of Medicine at Mount Sinai, New York, NY, United States; ^5^ Hasso Plattner Institute for Digital Health at Mount Sinai, Icahn School of Medicine at Mount Sinai, New York, NY, United States

**Keywords:** LLMS, large language model, artificial intelligence, psychiatry, generative pre-trained transformer (GPT)

## Abstract

**Background:**

With their unmatched ability to interpret and engage with human language and context, large language models (LLMs) hint at the potential to bridge AI and human cognitive processes. This review explores the current application of LLMs, such as ChatGPT, in the field of psychiatry.

**Methods:**

We followed PRISMA guidelines and searched through PubMed, Embase, Web of Science, and Scopus, up until March 2024.

**Results:**

From 771 retrieved articles, we included 16 that directly examine LLMs’ use in psychiatry. LLMs, particularly ChatGPT and GPT-4, showed diverse applications in clinical reasoning, social media, and education within psychiatry. They can assist in diagnosing mental health issues, managing depression, evaluating suicide risk, and supporting education in the field. However, our review also points out their limitations, such as difficulties with complex cases and potential underestimation of suicide risks.

**Conclusion:**

Early research in psychiatry reveals LLMs’ versatile applications, from diagnostic support to educational roles. Given the rapid pace of advancement, future investigations are poised to explore the extent to which these models might redefine traditional roles in mental health care.

## 1 Introduction

The integration of artificial intelligence (AI) into various healthcare sectors has brought transformative changes (1–3). Currently, Large Language Models (LLMs) like Chat Generative Pre-trained Transformer (ChatGPT) are at the forefront (2, 4, 5).

Advanced LLMs, such as GPT-4 and Claude Opus, possess an uncanny ability to understand and generate human-like text. This capacity indicates their potential to act as intermediaries between AI functionalities and the complexities of human cognition.

Unlike their broader application in healthcare, LLMs in psychiatry address unique challenges such as the need for personalized mental health interventions and the management of complex mental disorders (4, 6). Their capacity for human-like language generation and interaction is not just a technological advancement; it’s a critical tool in bridging the treatment gap in mental health, especially in under-resourced areas (4, 6–8).

In psychiatry, LLMs like ChatGPT can provide accessible mental health services, breaking down geographical, financial, or temporal barriers, which are particularly pronounced in mental health care (4, 9). For instance, ChatGPT can support therapists by offering tailored assistance during various treatment phases, from initial assessment to post-treatment recovery (10–12). This includes aiding in symptom management and encouraging healthy lifestyle changes pertinent to psychiatric care (10–14).

ChatGPT’s ability to provide preliminary mental health assessments and psychotherapeutic support is a notable advancement (15, 16). It can engage in meaningful conversations, offering companionship and empathetic responses, tailored to individual mental health needs (6, 10, 11, 13), a component that is essential in psychiatric therapy (17).

Despite these capabilities, currently, LLMs do not replace human therapists (8, 18, 19). Rather, the technology supplements existing care, enhancing the overall treatment process while acknowledging the value of human clinical judgment and therapeutic relationships (4, 8, 13).

As LLM technology advances rapidly, it holds the potential to alter traditional mental health care paradigms. This review aims to assess the current role of LLMs within psychiatry research, aiming to identify their strengths, limitations, and potential future applications. According to our knowledge, this is the first systematic review of the newer LLMs specifically within psychiatry.

## 2 Methods

### 2.1 Search strategy

The review was registered with the International Prospective Register of Systematic Reviews - PROSPERO (Registration code: CRD42024524035) We adhered to the Preferred Reporting Items for Systematic Reviews and Meta-Analyses (PRISMA) guidelines (20, 21).

A systematic search was conducted across key databases: PubMed, Embase, Web of Science, and Scopus, from December 2022 up until March 2024. We chose December 2022, as it was the date of introduction of chatGPT. We complemented the search via reference screening for any additional papers. We chose PubMed, Embase, Web of Science, and Scopus for their comprehensive coverage of medical and psychiatric literature.

Our search strategy combined specific keywords related to LLM, including ‘ChatGPT,’ ‘Artificial Intelligence,’ ‘Natural Language Processing,’ and ‘Large Language Models,’ with psychiatric terminology such as ‘Psychiatry’ and ‘Mental Health.’ Additionally, to refine our search, we incorporated keywords for the most relevant psychiatric diseases. These included terms like ‘Depression,’ ‘Anxiety Disorders,’ ‘Bipolar Disorder,’ ‘Schizophrenia,’ and others pertinent to our study’s scope.

Specific search strings for each database are detailed in the **Supplementary Materials**.

### 2.2 Study selection

The selection of studies was rigorously conducted by two independent reviewers, MO and EK. Inclusion criteria were set to original research articles that specifically examined the application of LLMs in psychiatric settings.

Eligible studies were required to present measurable outcomes related to psychiatric care, such as patient engagement, diagnostic accuracy, treatment adherence, or clinician efficiency.

We excluded review articles, case reports, conference abstracts without full texts, editorials, preprints, and studies not written in English.

MO and EK systematically evaluated each article against these criteria. In cases of disagreement or uncertainty regarding the eligibility of a particular study, the matter was resolved through discussion and, if necessary, consultation with additional researchers in our team to reach a consensus.

### 2.3 Data extraction

Data extraction was performed by two independent reviewers, MO and EK, using a structured template. Key information extracted included the study’s title, authors, publication year, study design, psychiatric condition or setting, the role of the LLM model, sample size, findings related to the effectiveness and impact of the model, and any noted conclusions and implications. In cases of discrepancy during the extraction process, issues were resolved through discussion and consultation with other researchers involved in the study.

### 2.4 Risk of bias

In our systematic review, we opted for a detailed approach instead of a standard risk of bias assessment, given the unique and diverse nature of the studies included. Each study is presented in a table highlighting its design and essential variables (**Table 1**).

**Table 1:** Summary of the included papers.

Author	Year	Model	Task	Application	Main Results
Liyanage et al. (22)	2023	GPT-3.5	Data augmentation for wellness dimension classification in Reddit posts	Social Media applications	ChatGPT models effectively augmented Reddit post data, significantly improving classification performance for wellness dimensions.
Hwang et al. (23)	2024	GPT-4	Generating psychodynamic formulations in psychiatry based on patient history	Clinical Reasoning	GPT-4 successfully created relevant and accurate psychodynamic formulations based on patient history.
Parker et al. (24)	2023	GPT-3	Providing information on bipolar disorder and generating creative content	Educational Therapeutic Interventions	GPT-3 provided basic material on bipolar disorders and creative song generation, but lacked depth for scientific review.
Heston T.F. et al. (25)	2023	GPT-3.5	Simulating depression scenarios and evaluating AI’s responses	Clinical Reasoning	ChatGPT-3.5 conversational agents recommended human support at critical points, highlighting the need for AI safety in mental health.
D’Souza et al. (26)	2023	GPT-3.5	Responding to psychiatric case vignettes with diagnostic and management strategies	Clinical Reasoning	ChatGPT 3.5 showed high competence in handling psychiatric case vignettes, with strong diagnostic and management strategy generation.
Levkovich et al. (27)	2023	GPT-3.5, GPT-4	Diagnosing and treating depression, comparing GPT models with primary care physicians	Clinical Reasoning	ChatGPT aligned with guidelines for depression management, contrasting with primary care physicians and showing no gender or socioeconomic biases.
Mazumdar et al. (28)	2023	GPT-3, BERT-large, MentalBERT, ClinicBERT, and PsychBERT	Classifying mental health disorders and generating explanations	Social Media applications	GPT-3 outperformed other models in classifying mental health disorders and generating explanations, showing promise for AI-IoMT deployment.
Sezgin et al. (29)	2023	GPT-4, LaMDA (using Bard)	Generating responses to postpartum depression questions and comparing accuracy	Educational Therapeutic Interventions	GPT-4 provided more clinically accurate responses to postpartum depression questions, surpassing other models and Google Search.
Elyoseph et al. (30)	2023	GPT-3.5	Evaluating emotional awareness compared to general population norms	Clinical Reasoning	ChatGPT showed higher emotional awareness compared to the general population and improved over time.
Elyoseph et al. (31)	2023	GPT-3.5	Assessing suicide risk in fictional scenarios and comparing to professional evaluations	Clinical Reasoning	ChatGPT underestimated suicide risks compared to mental health professionals, indicating the need for human judgment in complex assessments.
Levkovich et al. (32)	2023	GPT-3.5, GPT-4	Evaluating suicide risk assessments by GPT models and mental health professionals	Clinical Reasoning	GPT-4’s evaluations of suicide risk were similar to mental health professionals, though with some overestimations and underestimations.
Dergaa et al. (33)	2024	GPT-3.5	Simulated mental health assessments and interventions with ChatGPT	Clinical Reasoning	ChatGPT showed limitations in complex medical scenarios, underlining its unpreparedness for standalone use in mental health practice.
Spallek et al. (34)	2023	GPT-4	Providing educational material on mental health and substance use	Educational Therapeutic Interventions	GPT-4’s outputs were substandard compared to expert materials in terms of depth and adherence to communication guidelines.
Hadar-Shoval D et al. (35)	2023	GPT-3.5	Differentiating emotional responses in BPD and SPD scenarios using mentalizing abilities	Educational Therapeutic Interventions	ChatGPT effectively differentiated emotional responses in BPD and SPD scenarios, showing tailored mentalizing abilities.
Elyoseph et al. (36)	2024	GPT-3.5, GPT-4	Evaluating prognosis in depression compared to other LLMs and professionals	Clinical Reasoning	ChatGPT-3.5 showed a more pessimistic prognosis in depression compared to other LLMs and mental health professionals.
Li et al. (37)	2024	GPT-4, Bard and Llama-2	Evaluating performance on psychiatric licensing exams and diagnostics	Clinical Reasoning	GPT-4 outperformed other models in psychiatric diagnostics, closely matching the capabilities of human psychiatrists.

BPD, Borderline Personality Disorder; SPD, Schizoid Personality Disorder; GPT, Generative Pre-trained Transformer; AI, Artificial Intelligence; NLP, Natural Language Processing; EDA, Easy Data Augmentation; BT, Back Translation; PHQ-9, Patient Health Questionnaire-9; LEAS, Levels of Emotional Awareness Scale; AI-IoMT, Artificial Intelligence-Internet of Medical Things; GRADE, Grading of Recommendations, Assessment, Development, and Evaluations.

A second table catalogs the inherent limitations of each study, providing a transparent overview of potential biases and impacts on the results (**Table 2**).

**Table 2:** Summary of the studies designs, data, samples and limitations.

Author	Year	Study Design	Data type and Sample Size	Study Limitations
Liyanage et al. (22)	2023	Exploratory and Experimental Study	Real patient data from Reddit posts; 3,092 instances, post-augmentation 4,376 records	Limited generalizability due to the use of specific Reddit data and potential online discourse biases.
Hwang et al. (23)	2024	Exploratory and Experimental Study	Fictional patient data from published psychoanalytic literature; 1 detailed case	Reliance on fictional data from literature; lacks a comparator group for performance context.
Parker et al. (24)	2023	Exploratory and Experimental Study - Evaluative Research	NA	Clinical relevance limited by use of AI-generated responses and lack of real patient data.
Heston T.F. et al. (25)	2023	Observational Cross-Sectional Study	Fictional patient data; 25 conversational agents	Potential non-representativeness of simulations and small sample size of ChatGPT-3.5 agents.
D’Souza et al. (26)	2023	Experimental Study	Fictional patient data from clinical case vignettes; 100 cases	Fictional vignettes may not fully represent real-world psychiatric complexities; no comparator group.
Levkovich et al. (27)	2023	Cross-Sectional Analysis	Fictional patient data from clinical case vignettes; repeated multiple times for consistency	Hypothetical vignettes may lack real clinical scenario applicability; absence of patient demographics.
Mazumdar et al. (28)	2023	Retrospective and Prospective Analysis	Real patient data sourced from Reddit posts	Absence of demographic data and potential biases in retrospective data selection.
Sezgin et al. (29)	2023	Cross-Sectional Study	responses from LLMS and Google Search to postpartum depression questions; 14 questions	Lack of traditional participants; may not reflect clinical consultation complexity.
Elyoseph et al. (30)	2023	Comparative Prospective Study	Fictional scenarios from the LEAS; 750 participants	Fictional scenarios may not replicate real-world emotional challenges; comparison to general norms.
Elyoseph et al. (31)	2023	Comparative Retrospective Analysis	Fictional patient data; text vignettescompared to 379 professionals	Use of fictional vignettes; limited by specificity and generalizability.
Levkovich et al. (32)	2023	Prospective Vignette Study	Fictional patient data; text vignettescompared to 379 professionals	Hypothetical vignettes; focus on specific scenarios, not covering the full spectrum of risk factors.
Dergaa et al. (33)	2024	Prospective Simulated Interactions	Fictional patient data; 3 scenarios	Fictional scenarios; lacks complex real-patient interaction dynamics.
Spallek et al. (34)	2023	View Point and Case Study	Real-world queries from mental health and substance use portals; 10 queries	Small number of real-world queries; comparison to potentially biased expert materials.
Hadar-Shoval D et al. (35)	2023	Cross-Sectional Quantitative Analysis	Fictional patient data (BPD and SPD scenarios); AI-generated data	Fictional data limits real-world applicability; no comparator group.
Elyoseph et al. (36)	2024	Retrospective Comparative Analysis	Fictional patient data; text vignettescompared to 379 professionals	Use of fictional vignettes; focus on AI perspectives may have inherent biases.
Li et al. (37)	2024	Retrospective Analysis	Fictional patient data in exam and clinical scenario questions; 24 experienced psychiatrists	Fictional data for examination; limited comparison group size.

AI, Artificial Intelligence; NA, Not Available; NR, Not Reported; LEAS, Levels of Emotional Awareness.

**Figure 1:**
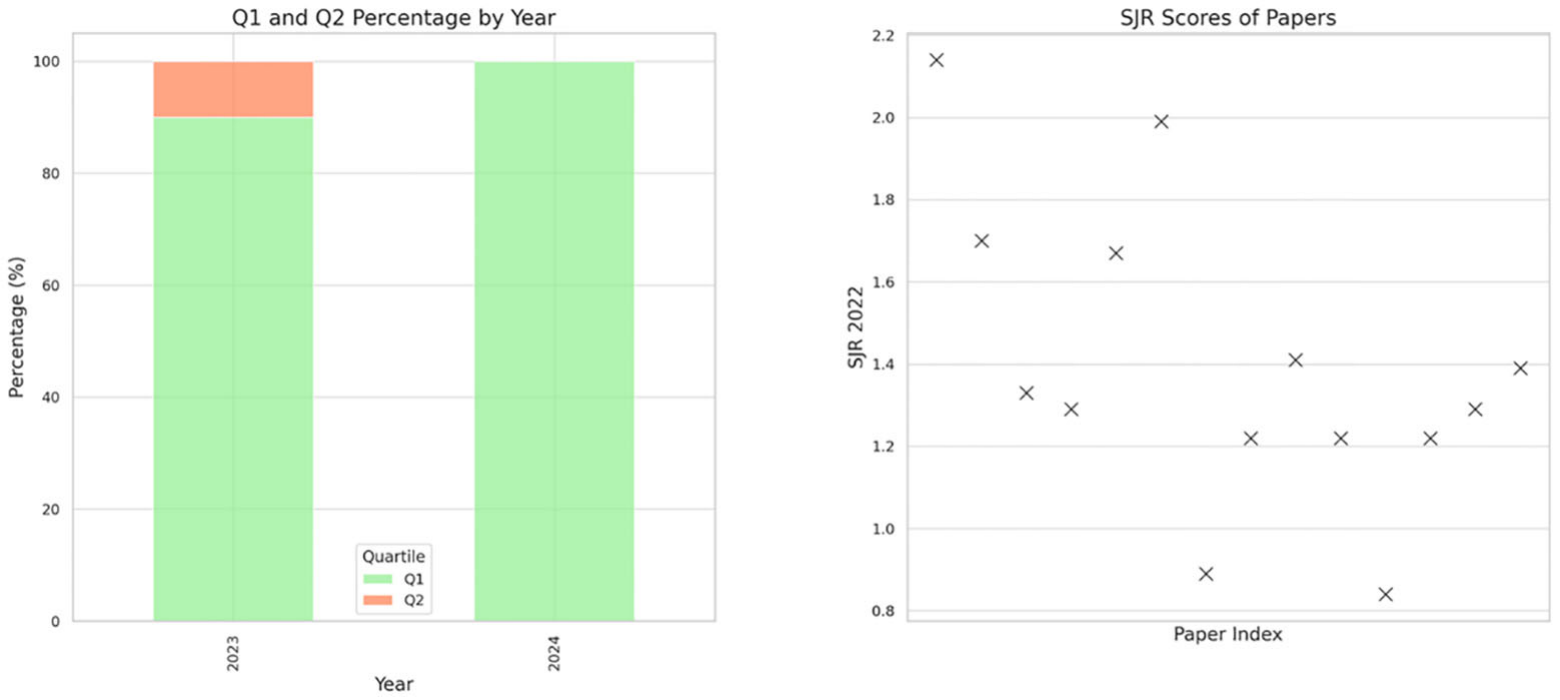
SJR scores and journal quartiles of the included studies.

Additionally, a figure illustrates the quartiles and SCImago Journal Rank scores of the journals where these studies were published, offering insight into their academic significance (**Figure 1**). This method ensures a clear, concise evaluation of the varied included papers.

## 3 Results

### 3.1 Search results and study selection

Our systematic search across PubMed, Embase, Web of Science, and Scopus yielded a total of 771 papers. The breakdown of the initial results was as follows: PubMed (186), Scopus (290), Embase (133), and Web of Science (162). After applying automated filters to exclude review articles, case reports, and other non-relevant document types, 454 articles remained. The removal of duplicates further reduced the pool to 288 articles.

**Figure 2:**
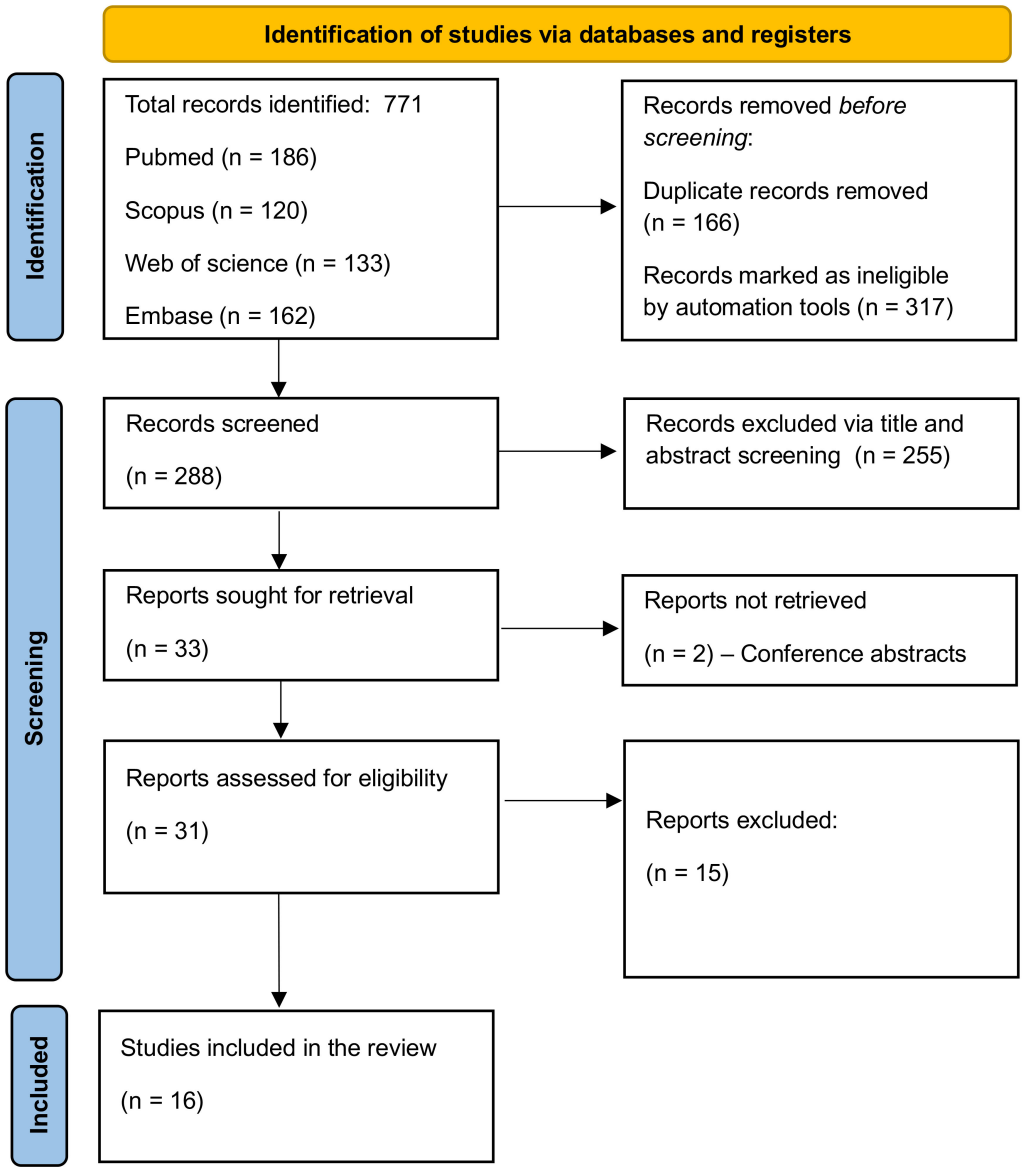
PRISMA flowchart.

Subsequent screening based on titles and abstracts led to the exclusion of 255 papers, primarily due to their irrelevance or lack of discussion on LLMs, leaving 33 articles for full-text evaluation. Upon detailed examination, 16 studies were found to meet our inclusion criteria and were thus selected for the final review (22–37). The process of study selection and the results at each stage are comprehensively illustrated in **Figure 2**, the PRISMA flowchart.

### 3.2 Overview of the included studies

Most of the studies in our review were published in Q1 journals, indicating a high level of influence in the field (**Figure 1**). The studies varied in their approach, using data ranging from real patient interactions on online platforms to simulated scenarios, and in scale, from individual case studies to large datasets.

**Figure 3:**
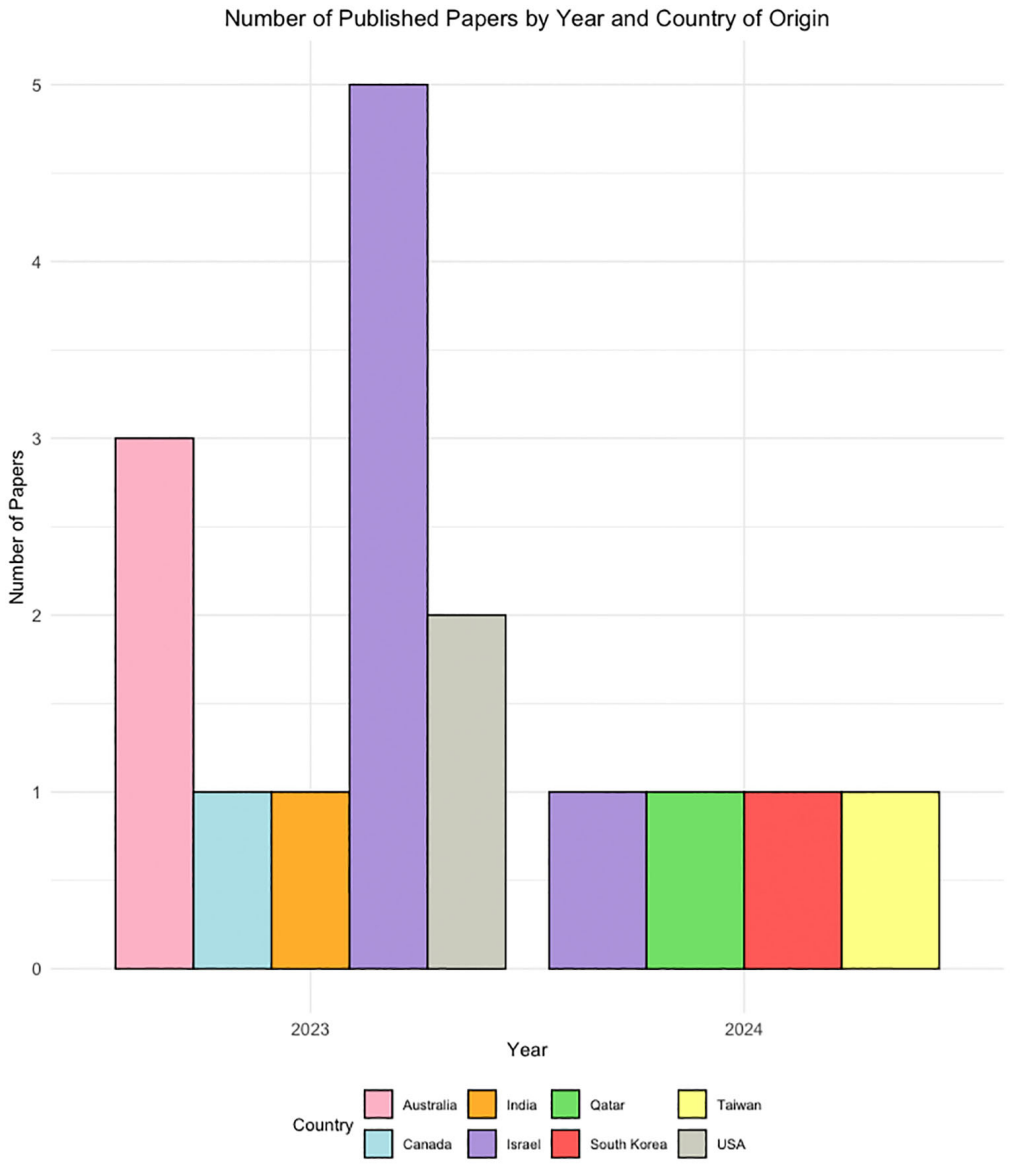
A demographic distribution graph showing the publication years and countries of origin for the included studies.

Most research focused on various versions of ChatGPT, including ChatGPT-3.5 and ChatGPT-4, with some comparing its performance to traditional methods or other LLMs. The applications of LLMs in these studies were diverse, covering aspects like mental health screening augmentation on social media, generating psychodynamic formulations, and assessing risks in psychiatric conditions. All of the included studies were published between 2023 and 2024, originating from 8 different countries with a relatively high number of papers from Israel (n = 5) (**Figure 3**).

In highlighting key studies, Liyanage et al. found that ChatGPT was effective in enhancing Reddit post analysis for wellness classification (22). Levkovich et al. observed ChatGPT’s unbiased approach in depression diagnosis, contrasting with biases noted in primary care physicians’ methods, especially due to gender and socioeconomic status (27). Additionally, Li et al. demonstrated GPT-4’s proficiency in psychiatric diagnostics, uniquely passing the Taiwanese Psychiatric Licensing Examination and paralleling experienced psychiatrists’ diagnostic abilities (37).

### 3.3 LLMs’ applications and limitations in mental health

We categorized the applications of LLM in the included studies into three main themes to provide a synthesized and comprehensive overview:

### 3.4 Applications

**Figure 4:**
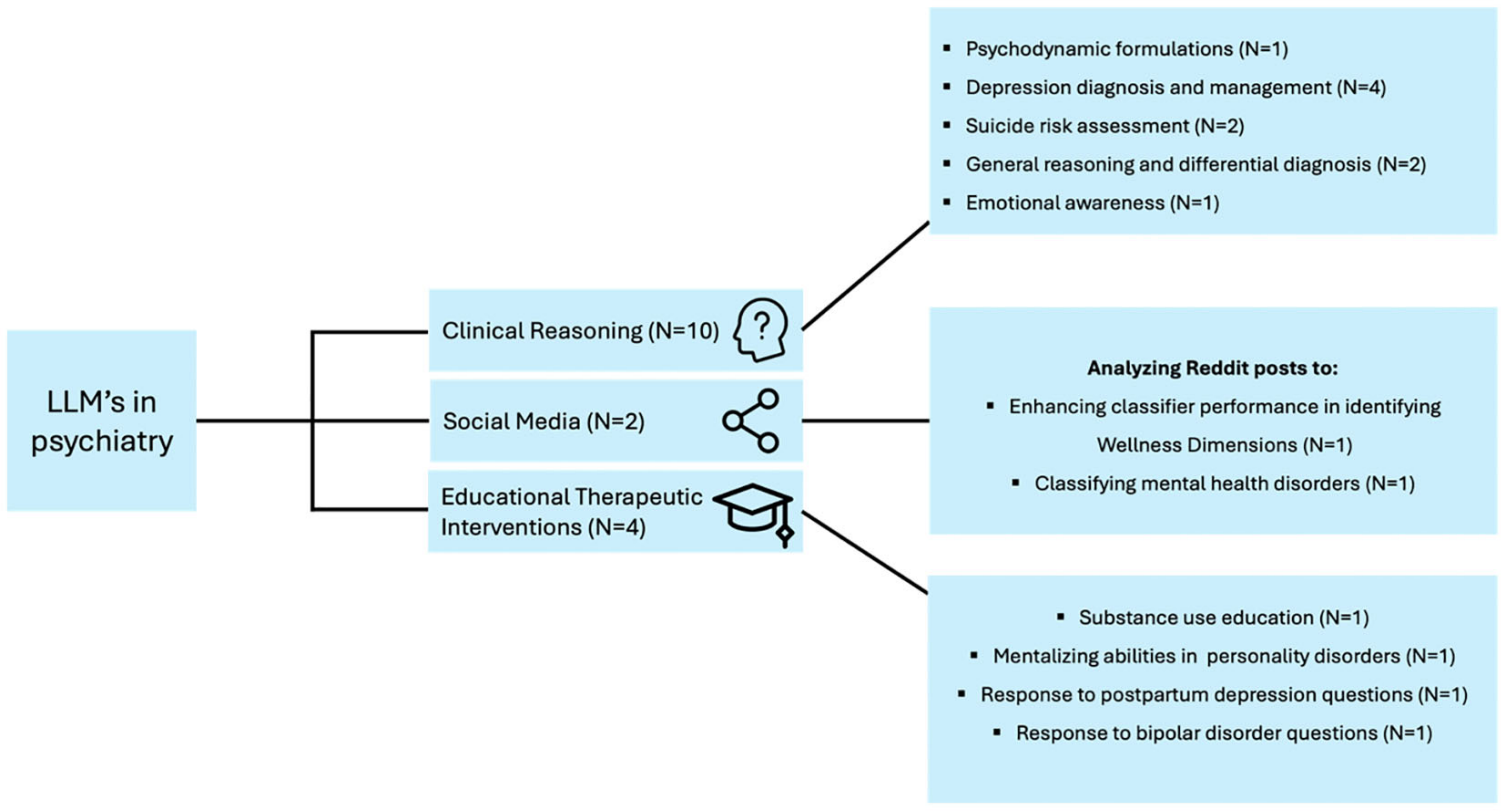
Applications and evaluations of LLMs in diverse domains of psychiatry.

We categorized the included studies into three broad categories based on their applications. Clinical reasoning encompasses studies where LLMs were used to generate psychodynamic formulations, simulate depression scenarios, handle psychiatric case vignettes, diagnose and treat depression, evaluate suicide risk, and assess emotional awareness and prognosis in depression. Social media applications include studies that leveraged LLMs for data augmentation and classifying mental health disorders from Reddit posts. Educational therapeutic interventions cover studies focused on providing educational material on mental health topics, generating creative content, and differentiating emotional patient responses in personality disorder scenarios (**Table 1**, **Figure 4**).

#### 3.4.1 Clinical reasoning

- Hwang et al. demonstrated ChatGPT’s ability in generating psychodynamic formulations, indicating potential in clinical psychiatry with statistical significance (Kendall’s W = 0.728, p = 0.012) (23).
- Levkovich et al. (2023) compared ChatGPT’s recommendations for depression treatment against primary care physicians, finding ChatGPT more aligned with accepted guidelines, particularly for mild depression (27).
- Levkovich et al. and Elyoseph et al. (2023) assessed ChatGPT’s performance in assessing suicide risk. The study by Levkovich et al. highlighted that ChatGPT tended to underestimate risks when compared to mental health professionals, particularly in scenarios with high perceived burdensomeness and feeling of thwarted belongingness (32). The study be Elyoseph, found that while GPT-4’s evaluations were similar to mental health professionals, ChatGPT-3.5 often underestimated suicide risk (31).
- D’Souza et al. evaluated ChatGPT’s response to psychiatric case vignettes, where it received high ratings, especially in generating management strategies for conditions like anxiety and depression (26).
- Li et al. demonstrated ChatGPT GPT-4’s capabilities in the Taiwanese Psychiatric Licensing Examination and psychiatric diagnostics, closely approximating the performance of experienced psychiatrists (37). ChatGPT outperformed the two LLMs, Bard and Llama-2.
- Heston T.F. et al. Evaluated ChatGPT-3.5’s responses in depression simulations. AI typically recommended human support at moderate depression levels (PHQ-9 score of 12) and insisted on human intervention at severe levels (score of 25) (25).
- Dergaa et al. critically assessed ChatGPT’s effectiveness in mental health assessments, particularly highlighting its inadequacy in dealing with complex situations, such as nuanced cases of postpartum depression requiring detailed clinical judgment, suggesting limitations in its current readiness for broader clinical use (33).
- Elyoseph et al. (2024) provided a comparative analysis of depression prognosis from the perspectives of AI models, mental health professionals, and the general public. The study revealed notable differences in long-term outcome predictions. AI models, including ChatGPT, showed variability in prognostic outlooks, with ChatGPT-3.5 often presenting a more pessimistic view compared to other AI models and human evaluation (36).
- Elyoseph et al. (2023) investigated ChatGPT’s emotional awareness using the Levels of Emotional Awareness Scale (LEAS). ChatGPT scored significantly higher than the general population, indicating a high level of emotional understanding (30).

#### 3.4.2 Social media applications

- Liyanage et al. used ChatGPT models to augment data from Reddit posts, enhancing classifier performance in identifying Wellness Dimensions. This resulted in improvements in the F-score of up to 13.11% (22).
- Mazumdar et al. applied GPT-3 in classifying mental health disorders from Reddit data, achieving an accuracy of around 87% and demonstrating its effectiveness in explanation generation (28). GPT-3 demonstrated superior performance in classifying mental health disorders and generating explanations, outperforming traditional models like LIME and SHAP.

**Figure 5:**
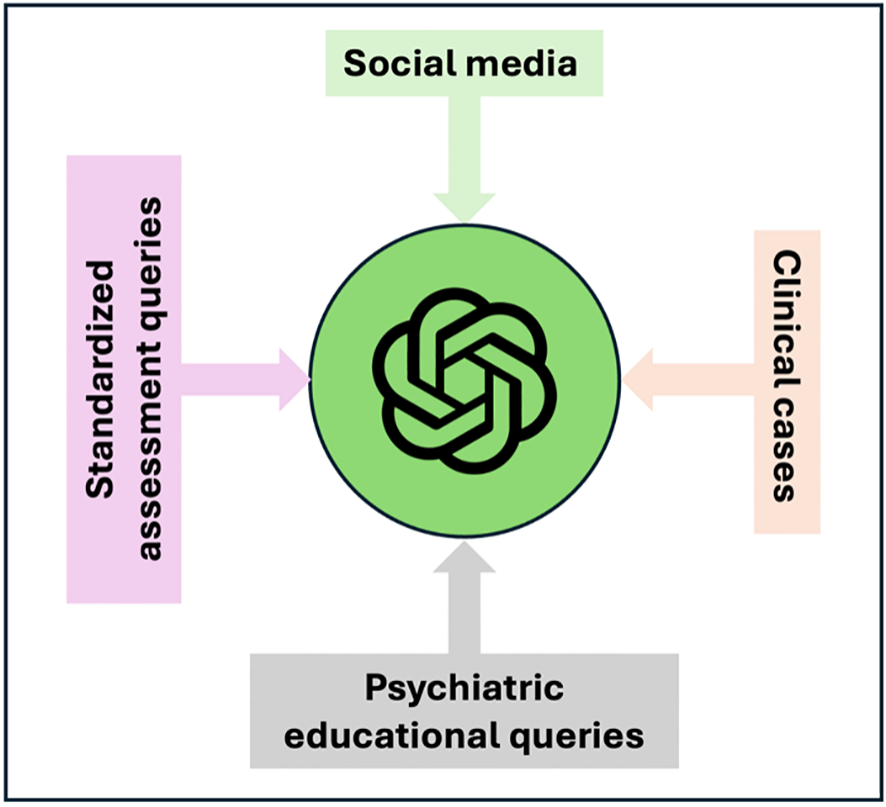
Data Input Spectrum for GPT in Psychiatric Applications.

**Figure 5** presents the different types of data inputs for GPT in the current applications for the field of psychiatry.

#### 3.4.3 Educational therapeutic interventions

- Spallek et al. examined ChatGPT’s application in mental health and substance use education, finding its outputs to be substandard compared to expert materials. However, when prompts where carefully engineered, the outputs were better aligned with communication guidelines (34).
- Hadar-Shoval D et al., explored ChatGPT’s ability to understand mental state in personality disorders (35), and Sezgin et al. (29), assessed responses to postpartum depression questions Both studies reflect ChatGPT’s utility both as an educational resource and for offering preliminary therapeutic advice. Sezgin et al. (29) showed that GPT-4 demonstrated generally higher quality, more clinically accurate responses compared to Bard and Google Search.
- Parker et al. ChatGPT-3.5 was used to respond to clinically relevant questions about bipolar disorder and to generate songs related to bipolar disorder, testing both its factual knowledge and creativity. The study highlighted its utility in providing basic information, but also its limitations in citing current, accurate references (24).

The studies collectively highlight that while LLMs are generally reliable, they exhibit variability in handling false positives and false negatives across different psychiatric applications. For example, Hwang et al. demonstrated that ChatGPT produced reliable psychodynamic formulations with minimal false positives (Kendall’s W = 0.728, p = 0.012) (23). Conversely, Levkovich et al. and Elyoseph et al. found that ChatGPT versions often underestimated suicide risks, indicating a tendency towards false negatives (31, 32). Specifically, ChatGPT-3.5 underestimated the risk of suicide attempts with an average Z score of -0.83 compared to mental health professionals (Z score +0.01) (31, 32). Liyanage et al. showed that data augmentation with ChatGPT models significantly improved classifier performance for wellness dimensions in Reddit posts, reducing both false positives and false negatives, with an improvement in F-score by up to 13.11% and Matthew’s Correlation Coefficient by up to 15.95% (22).

Additional data in the **Supplementary Materials** includes demographics, journals of the included papers, SCImago Journal Rank (SJR) 2022 data for these publications, and specific performance metrics for the models across various applications, detailed in **Tables S1, S2** in the **Supplementary Material**.

### 3.5 Safety and limitations

Concerns regarding safety and limitations in LLMs clinical applications emerge as critical themes. For instance, Heston T.F. et al. observed that ChatGPT-3.5 recommended human support at moderate depression levels but only insisted on human intervention at severe levels, underscoring the need for cautious application in high-risk scenarios (25).

Elyoseph et al. highlighted that ChatGPT consistently underestimated suicide risks compared to mental health professionals, especially in scenarios with high perceived burdensomeness and thwarted belongingness (31).

**Figure 6:**
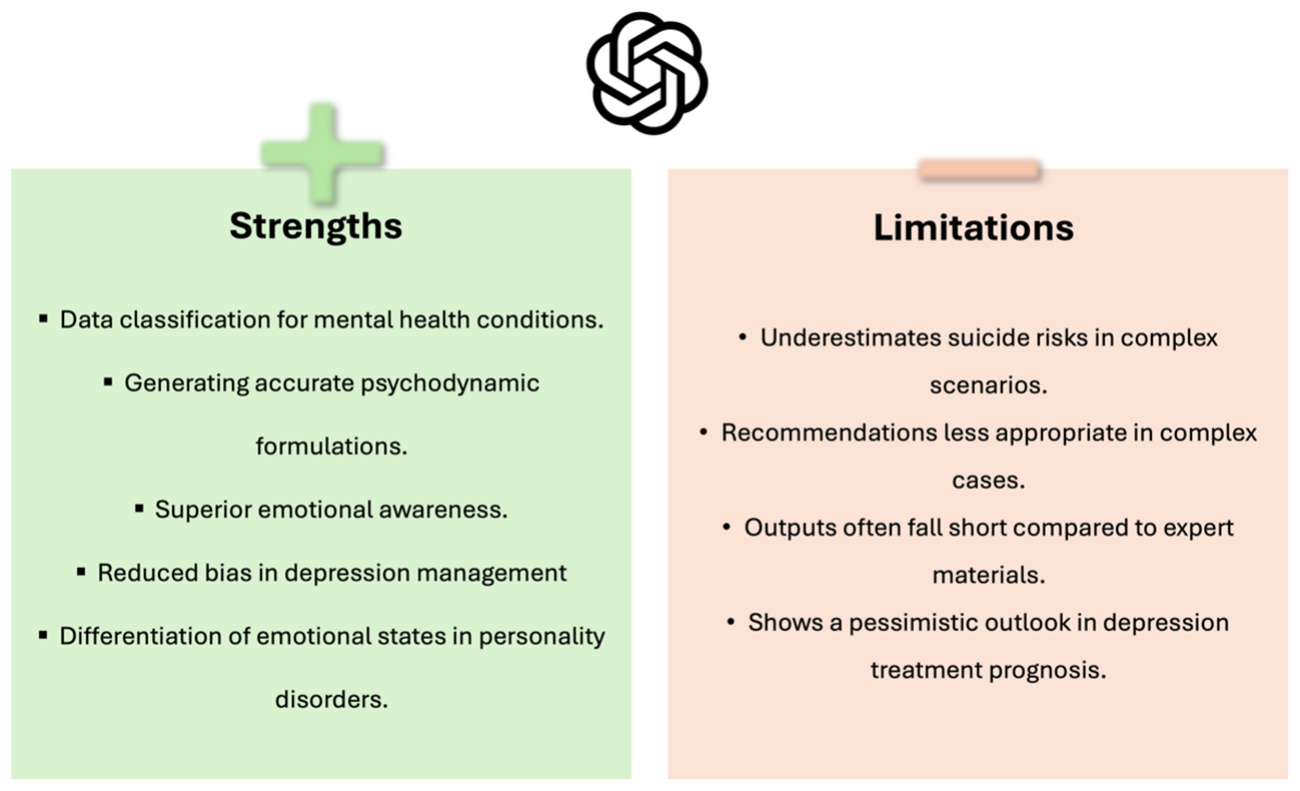
Strengths and limitations of GPT’s current applications in psychiatry practice.

Dergaa et al. concluded that ChatGPT, as of July 2023, was not ready for mental health assessment and intervention roles, showing limitations in complex case management (33). This suggest that while ChatGPT shows promise, it is not without significant risks and limitations, particularly in handling complex and sensitive mental health scenarios (**Table 2**, **Figure 6**).

## 4 Discussion

Our findings demonstrate LLMs, especially ChatGPT and GPT-4, potential as a valuable tool in psychiatry, offering diverse applications from clinical support to educational roles. Studies like Liyanage et al. and Mazumdar et al. showcased its efficacy in data augmentation and mental health disorder classification (22, 28). Others, such as Hwang et al. and Levkovich et al. (2023), highlighted its capabilities in clinical settings, including diagnosis and risk assessment (23, 27). Overall, GPT emerged as the most used and studied LLM in the field of psychiatry.

In Clinical Reasoning, LLMs like GPT-4 showed effectiveness in generating psychodynamic formulations, accurately diagnosing and treating psychiatric conditions, and performing psychiatric diagnostics on par with human professionals. However, their capability in assessing and managing suicide risk was limited, often underestimating risks, which underscores the need for human oversight. Additionally, LLMs demonstrated higher emotional awareness compared to the general population but presented a more pessimistic prognosis in depression cases. In Social Media Applications, LLMs enhanced data augmentation and significantly improved classification performance for wellness dimensions in social media posts, outperforming other models in classifying mental health disorders. Educational Therapeutic Interventions revealed that while LLMs can generate educational content and creative therapeutic materials, their outputs often lack the depth and adherence to guidelines found in expert-developed materials (**Table 3**).

**Table 3:** High-level research questions and key findings across studies.

Clinical Reasoning		
High-Level Research Question	Studies	Key Findings
Can LLMs accurately diagnose and treat psychiatric conditions?	Levkovich et al. (2023), D’Souza et al. (2023) (27, 36)	ChatGPT aligned with guidelines for depression management, showing high competence in handling psychiatric case vignettes.
What is the capability of LLMs in assessing and managing suicide risk?	Elyoseph et al. (2024), Levkovich et al. (2023) (31, 32)	ChatGPT often underestimated suicide risks, indicating the need for human judgment in complex assessments.
How do LLMs perform in psychiatric clinical diagnostics?	Li et al. (2024) (37)	GPT-4 outperformed other models in psychiatric diagnostics, closely matching the capabilities of human psychiatrists.

Social Media Applications		
High-Level Research Question	Studies	Key Findings
How effective are LLMs in classifying mental health disorders from social media data?	Mazumdar et al. (2023) (28)	GPT-3 outperformed other models in classifying mental health disorders and generating explanations.

Educational Therapeutic Interventions		
High-Level Research Question	Studies	Key Findings
What is the efficacy of LLMs in generating educational content for mental health?	Spallek et al. (2023) (34)	GPT-4’s educational outputs were substandard compared to expert materials, while GPT-3 provided basic information on bipolar disorder but lacked scientific depth.
How accurately can LLMs respond to clinical questions about specific conditions?	Sezgin et al. (2023) (29)	GPT-4 provided more clinically accurate responses to postpartum depression questions compared to other models and Google Search.

However, concerns about its limitations and safety in clinical scenarios were evident, as seen in studies by Elyoseph et al. (2023) and Dergaa et al., indicating that while ChatGPT holds promise, its integration into clinical psychiatry must be approached with caution (31, 33).

The potential and efficacy of AI, particularly LLMs, in psychiatry are highlighted by our review, showing its capability to streamline care, lower barriers, and reduce costs in mental health services (38). Studies like Liyanage et al. and Hwang et al. illustrate ChatGPT’s diverse applications, from clinical data analysis to formulating psychodynamic profiles, which contribute to a more efficient, accessible, and versatile approach in mental healthcare (8, 19, 22, 23). Moreover, in light of the COVID-19 pandemic’s impact on mental health and the growing demand for digital interventions, Mitsea et al.’s research highlights the significant role of AI in enhancing digitally assisted mindfulness training for self-regulation and mental well-being, further enriching the scope of AI applications in mental healthcare (39).

When compared with humans and other LLMs, ChatGPT consistently adheres to clinical guidelines, as shown in Levkovich et al.’s study (27). Furthermore, ChatGPT often surpasses other models in tasks like psychiatric diagnostics, as demonstrated by Li et al. (37).

These studies collectively underscore the utility of current LLMs, particularly ChatGPT and GPT-4, in psychiatry, demonstrating promise across various domains. While serving as a complementary tool to human expertise, especially in complex psychiatric scenarios (4, 6, 11, 19), LLMs are poised for deeper integration into mental health care. This evolution is propelled by rapid technological advancements and significant financial investments since the watershed moment of ChatGPT introduction, late 2022. Future research should closely monitor this integration, exploring how LLMs not only supplement but also augment human expertise in psychiatry.

GPT-4 generally shows higher interpretability due to more transparent decision-making processes (39, 40). Advanced models like GPT-4 also typically incorporate better security measures and stricter privacy protocols, essential for handling sensitive psychiatric data (41). Regarding computational resources, GPT-4’s training involves significant resources, such as 8 TPU Pods and 512GB of RAM, while its inference requires 2 TPU Pods and 64GB of RAM (42). This suggests a need for robust infrastructure for real-world applications. Nonetheless, the internet interface is widely available and easily usable, in addition to the API usage for streamlining different applications more efficiently (42). This could imply a future where these models can be relatively easily implemented and used. However, ethical and privacy restrictions need further research.

Our review has limitations. The absence of a formal risk of bias assessment, due to the unique nature of the included studies, is a notable drawback. Additionally, the reliance on studies that did not use real patient data as well as the heterogeneity in study designs could affect the generalizability of our findings. Moreover, the diversity of methods and tasks in the included studies prohibited us from performing a meta-analysis. It should also be mentioned that all studies were retrospective in nature. Future directions should include prospective, real-world evidence studies, that could cement the utility of LLM in the psychiatry field.

In conclusion, our review highlights the varied performance of LLMs like ChatGPT in mental health applications. In clinical reasoning, ChatGPT demonstrated strong potential, generating psychodynamic formulations with high interrater agreement and providing depression treatment recommendations closely aligned with guidelines, particularly for mild depression. However, it often underestimated suicide risk in high-risk scenarios. In social media applications, ChatGPT models enhanced classifier performance for wellness dimensions on platforms like Reddit, with F-score improvements up to 13.11% and Matthew’s Correlation Coefficient increases by 15.95%. In classifying mental health disorders, GPT-3 achieved around 87% accuracy and strong ROUGE-L scores. For educational and therapeutic interventions, ChatGPT’s outputs improved significantly with carefully engineered prompts, aligning better with communication guidelines and readability standards. However, it struggled in complex clinical scenarios, revealing limitations in its readiness for broader clinical use.

## Data availability statement

The original contributions presented in the study are included in the article/**Supplementary Material**, further inquiries can be directed to the corresponding author/s.

## Author contributions

MO: Conceptualization, Data curation, Formal analysis, Funding acquisition, Investigation, Methodology, Project administration, Resources, Software, Supervision, Validation, Visualization, Writing – original draft, Writing – review & editing. SS: Validation, Writing – review & editing. AC: Investigation, Supervision, Writing – review & editing. IL: Formal analysis, Investigation, Methodology, Validation, Writing – review & editing. GN: Conceptualization, Supervision, Writing – review & editing. EK: Conceptualization, Formal analysis, Investigation, Methodology, Project administration, Supervision, Writing – original draft, Writing – review & editing.
