# Supplementary material for: Applications of large language models in psychiatry: a systematic review
Source: Front Psychiatry. 2024 Jun 24;15:1422807. doi: 10.3389/fpsyt.2024.1422807 (PMC11228775; doi:10.3389/fpsyt.2024.1422807)
Supplement: Supplementary file 1 [file DataSheet_1.docx]

**Supplementary material**

Specific Boolean strings for each database:

Pubmed

("ChatGPT" OR "GPT-3" OR "OpenAI language model" OR "AI Chatbot" OR "Artificial Intelligence in psychiatry" OR "LLM" OR "large language model") AND ("psychiatry" OR "mental health" OR "depression" OR "anxiety" OR "bipolar disorder" OR "schizophrenia" OR "PTSD" OR "obsessive-compulsive disorder" OR "eating disorder" OR "personality disorder")

Scopus

( TITLE-ABS-KEY ( "ChatGPT" OR "GPT-3" OR "OpenAI language model" OR "AI Chatbot" OR "Artificial Intelligence in psychiatry" OR "LLM" OR "large language model" ) AND TITLE-ABS-KEY ( "psychiatry" OR "mental health" OR "depression" OR "anxiety" OR "bipolar disorder" OR "schizophrenia" OR "PTSD" OR "obsessive-compulsive disorder" OR "eating disorder" OR "personality disorder" ) )

Embase

('ChatGPT'/exp OR 'GPT-3'/exp OR 'OpenAI language model'/exp OR 'AI Chatbot'/exp OR 'Artificial Intelligence in psychiatry'/exp OR 'LLM'/exp OR 'large language model'/exp) AND ('psychiatry'/exp OR 'mental health'/exp OR 'depression'/exp OR 'anxiety'/exp OR 'bipolar disorder'/exp OR 'schizophrenia'/exp OR 'PTSD'/exp OR 'obsessive-compulsive disorder'/exp OR 'eating disorder'/exp OR 'personality disorder'/exp)

Web of science

(TS=("ChatGPT" OR "GPT-3" OR "OpenAI language model" OR "AI Chatbot" OR "Artificial Intelligence in psychiatry" OR "LLM" OR "large language model") AND TS=("psychiatry" OR "mental health" OR "depression" OR "anxiety" OR "bipolar disorder" OR "schizophrenia" OR "PTSD" OR "obsessive-compulsive disorder" OR "eating disorder" OR "personality disorder"))

**Supplementary Table S1: Additional Study Details**

| **N** | **Author** | **Year** | **Journal** | **SJR 2022** | **Q** | **Data Type** | **Demographics** | **Comparator** | **Outcome Measures** | **Performance Metrics** |
| --- | --- | --- | --- | --- | --- | --- | --- | --- | --- | --- |
| 1 | Liyanage et al.(34) | 2023 | Book chapter | NA | NA | Real patient data from Reddit | Not Reported (NR) | Traditional NLP augmentation methods | Efficacy of data augmentation in wellness dimension classification | F-score and Matthew's Correlation Coefficient improvement; ROUGE scores for augmented data |
| 2 | Hwang et al.(35) | 2024 | Psychiatry research | 2.14 | 1 | Fictional patient data | Artist with panic attacks and depression history | No specific comparator group | Accuracy of psychodynamic formulations generated by ChatGPT | Kendall’s W = 0.728 for interrater agreement |
| 3 | Liyanage et al.(34) | 2023 | Bipolar disorders | 1.7 | 1 | AI-generated responses | NA | No direct comparison to other LLMs or psychiatrists | Accuracy and comprehensiveness of responses on bipolar disorder | Not Reported (NR) |
| 4 | Hwang et al.(35) | 2023 | cuerues | NA | NA | Fictional patient data for simulations | NA | No direct comparator group | AI response patterns in depression and suicidality scenarios | Initial referral and shutdown corresponding with PHQ-9 scores |
| 5 | Liyanage et al.(34) | 2023 | Asian Journal of Psychiatry | 1.33 | 1 | Fictional patient vignettes | NA | No comparator group | ChatGPT 3.5's simulation of psychiatric diagnosis and management strategies | Graded performance in handling vignettes |
| 6 | Hwang et al.(35) | 2023 | Family medicine and community health | 1.29 | 1 | Hypothetical patient vignettes | NA | Primary care physicians | Treatment strategy recommendations for depression | Statistical comparison of treatment recommendations with primary care physicians |
| 7 | Liyanage et al.(34) | 2023 | IEEE journal of biomedical and health informatics | 1.67 | 1 | Real patient data from Reddit | NR | BERT-large, MentalBERT, ClinicBERT, PsychBERT | Effectiveness in classifying mental health disorders and generating explanations | Classification accuracy and ROUGE-L scores for explanations |
| 8 | Hwang et al.(35) | 2023 | Medical Internet research | 1.99 | 1 | Responses to FAQ | NA | LaMDA (using Bard), Google Search | Quality of responses to postpartum depression questions | GRADE-informed quality assessment scale; mean quality rating for ChatGPT |
| 9 | Liyanage et al.(34) | 2023 | Frontiers in psychology | 0.89 | 2 | Fictional scenarios from LEAS | General French population, range 17-84 years | General population norms from a 2013 study | Emotional awareness evaluations | Z scores comparing ChatGPT's LEAS performance with human norms |
| 10 | Hwang et al.(35) | 2023 | Frontiers in psychiatry | 1.22 | 1 | Fictional text vignettes | 37-year-old single woman | Mental health professionals | Suicide risk assessment considering perceived burdensomeness and thwarted belongingness | Comparisons of ChatGPT's ratings of psychache, suicidal ideation, risk of suicide attempt, and resilience with mental health professionals |
| 11 | Liyanage et al.(34) | 2023 | JMIR mental health | 1.41 | 1 | Hypothetical patient vignettes | Mental health professionals, demographics reported | Mental health professionals | Suicide risk assessment vignettes | Comparison of ChatGPT's assessments of psychache, suicidal ideation, risk of suicide attempt, and resilience with professionals |
| 12 | Hwang et al.(35) | 2024 | Frontiers in psychiatry | 1.22 | 1 | Fictional patient scenarios | Imaginary patients including students and post-partum women | No explicit comparator group | Effectiveness in mental health assessment and interventions | Not Reported (NR) |
| 13 | Liyanage et al.(34) | 2023 | JMIR medical education | 0.84 | 1 | Real-world user queries | NA | Expert-developed educational materials | Quality and reliability of educational health materials | Sydney Health Literacy Lab tool for readability and text complexity; adherence to communication guidelines |
| 14 | Hwang et al.(35) | 2023 | Frontiers in psychiatry | 1.22 | 1 | AI-generated data | NA | No direct comparator group | Emotional responses in BPD and SPD scenarios | LEAS scores for emotional awareness in BPD vs SPD scenarios |
| 15 | Liyanage et al.(34) | 2024 | Family medicine and community health | 1.29 | 1 | Fictional patient vignettes | Mental health nurses, psychiatrists, GPs, clinical psychologists, general public | Mental health professionals and the general public | Prognosis and long-term outcomes in depression | ANOVA for negative and positive long-term outcomes predicted by LLMs |
| 16 | Hwang et al.(35) | 2024 | Psychiatry and Clinical Neurosciences | 1.39 | 1 | Fictional patient data for examination and clinical scenarios | NA | Other LLMs (Bard and Llama-2) and human psychiatrists | Psychiatric licensing examination performance and clinical diagnostic accuracy | Comparison of examination scores and clinical scenario performance with psychiatrists |

Abbreviations: AI: Artificial Intelligence | ANOVA: Analysis of Variance | BPD: Borderline Personality Disorder | BT: Back Translation | EDA: Easy Data Augmentation | GPs: General Practitioners | GRADE: Grading of Recommendations, Assessment, Development, and Evaluations | GPT: Generative Pre-trained Transformer | JMIR: Journal of Medical Internet Research | LEAS: Levels of Emotional Awareness Scale | LLM: Large Language Model | NA: Not Available | NR: Not Reported | NLP: Natural Language Processing | PHQ-9: Patient Health Questionnaire-9 | SJR: SCImago Journal Rank | SPD: Schizoid Personality Disorder.

**Supplementary Table S2: SJR scores and journal quartiles of the included studies**

| **Title** | **Year** | **SJR 2022** | **Q** |
| --- | --- | --- | --- |
| Augmenting Reddit Posts to Determine Wellness Dimensions impacting Mental Health | 2023 | NA | NA |
| Assessing the potential of ChatGPT for psychodynamic formulations in psychiatry- An exploratory study | 2024 | 2.14 | 1 |
| A chat about bipolar disorder | 2023 | 1.7 | 1 |
| Safety of Large Language Models in Addressing Depression | 2023 | NA | NA |
| Appraising the performance of ChatGPT in psychiatry using 100 clinical case vignettes | 2023 | 1.33 | 1 |
| Identifying depression and its determinants upon initiating treatment- ChatGPT versus primary care physicians | 2023 | 1.29 | 1 |
| GPTFX- A Novel GPT-3 Based Framework for Mental Health Detection and Explanations | 2023 | 1.67 | 1 |
| Clinical Accuracy of Large Language Models and Google Search Responses to Postpartum Depression Questions- Cross-Sectional Study | 2023 | 1.99 | 1 |
| ChatGPT outperforms humans in emotional awareness evaluations | 2023 | 0.89 | 2 |
| Beyond human expertise- the promise and limitations of ChatGPT in suicide risk assessment | 2023 | 1.22 | 1 |
| Suicide Risk Assessments Through the Eyes of ChatGPT-3.5 Versus ChatGPT-4- Vignette Study | 2023 | 1.41 | 1 |
| ChatGPT is not ready yet for use in providing mental health assessment and interventions | 2024 | 1.22 | 1 |
| Can we use ChatGPT for Mental Health and Substance Use Education? Examining Its Quality and Potential Harms | 2023 | 0.84 | 1 |
| The plasticity of ChatGPT's mentalizing abilities- personalization for personality structures | 2023 | 1.22 | 1 |
| Assessing prognosis in depression- comparing perspectives of AI models, mental health professionals and the general public | 2024 | 1.29 | 1 |
| Comparing the performance of ChatGPT GPT-4, Bard, and Llama-2 in the Taiwan Psychiatric Licensing Examination and in differential diagnosis with multi-center psychiatrists | 2024 | 1.39 | 1 |
